# Bi-Chloride of Mercury in Diphtheria

**Published:** 1883-12-20

**Authors:** Madison Reece

**Affiliations:** Abingdon, Ill.


### BI-CHLORIDE OF MERCURY IN DIPHTHERIA.

BY MADISON REECE, M.D., ABINGDON, ILL

During the past two and a half years, I have used, exclusively, in the treatment of diphtheria, the bi-chloride of mercury in large and frequent doses. My attention was called to its use by reading the address of Dr. Wm. Pepper, chairman of the Section of Practical Medicine, before the American Medical Association for the year 1881. The statements therein made interested me to such an extent that, having on hand two cases of this disease of a malignant form, I determined to try its efficacy.

Up to this time I had found (as who has not?) true diphtheria one of the most fatal forms of the disease that could be encountered. I had used the usual remedies, so far as I could observe, without any effect upon the progress of the disease, and had arrived at the conclusion that in the worst forms of the disease the patient would die with or without treatment, but since adopting the method of treatment to be described, I have not felt the same anxiety as formerly, when called to a case.

To this date thirty-five cases have been treated in this way, with three deaths. Two of these deaths were the first cases referred to above, and although they ended fatally, I was thoroughly convinced that the remedy had special power to combat the disease, and I now believe that with my present experience in the use of this remedy, I could have saved one, if not both of these patients.

My method of preparing this medicine is to dissolve one grain of the bi-chloride in four ounces of rain-water; then, if the patient is old enough to gargle and rinse the throat and mouth, he is to do so every two hours, and immediately afterwards to take a teaspoonful internally. If the disease be of a severe form, it should be administered in this way every hour. The above dose is calulated for a child of five years of age. I have often used the same amount for a child of two years of age.

It will be observed within fifteen or twenty hours that the exudations on the tonsils and palate will begin to fade away and in a few hours more rapidly disappear. If then, unfortunately, as I found by experience in my early use of the remedy, the medicine be discontinued, the exudation will rapidly re-appear, to be again dispersed by a return to the treatment, so that it is necessary to continue for a week, or even a longer time, the use of the medicine, not in such large and frequent doses, for it is observed that as soon as the patient shows signs of becoming better, the effects of the bi-chloride are shown by nausea, or vomiting, or purging. But so long as the system seems to be laboring under the diphtheritic poison, these effects are not manifested.

We shall not attempt to give the *rationale* of the action of this medicine, but will only call attention to the fact that it belongs to that class of remedies which is rich in chlorine, and to which physicians have resorted for many years in the treatment of this affection, such as the tr. of the chloride of iron, chlorine water, chlorate of potassium, and here the bi-chloride of mercury. Also in view of the strong germicidal qualities of this substance,, as recently demonstrated by Dr. Sternberg, we may reasonably suppose it has a destructive effect on the bacteria that swarm in the exudation in the throat and surrounding structures.

To show that this remedy in diphtheria seems to be appreciated abroad, I quote from Dr. Sternberg's article in the April number of the American Journal of the Medical Sciences, page 337:

"A medical friend who has just returned from Vienna, informs me that mercuric bi-chloride is at present the favorite remedy in that city for diphtheria.

My friends and neighbors, Dr. H. Judd, of Galesburg, and Dr. W. G. Piersol, of Hermon, have used this remedy in their practice with the most satisfactory results.

In conclusion, I would request those who may make a trial of this treatment to communicate the result to the Journal, or if not wishing to do so, to the writer.—*Jour. Ame. Med. Assn.*

